# Preface to the Special Issue on Assessment and Effectiveness of Driver Monitoring Systems

**DOI:** 10.1177/00187208231206073

**Published:** 2023-11-13

**Authors:** Francesco N. Biondi, William J. Horrey, Birsen Donmez

**Affiliations:** 1 8637 University of Windsor, Windsor, ON, Canada; 2 172839 University of Utah, Salt Lake City, UT, USA; 3 14579 AAA Foundation for Traffic Safety, Washington, DC, USA; 4 7938 University of Toronto, Toronto, ON, Canada

**Keywords:** automation, driver monitoring systems, driver behavior, alertness, distraction

## Abstract

With vehicle automation becoming more commonplace, the role of the human driver is shifting from that of system operator to that of system supervisor. With this shift comes the risk of drivers becoming more disengaged from the task of supervising the system functioning, thus increasing the need for technology to keep drivers alert. This special issue includes the most up-to-date research on how drivers use vehicle automation, and the safety risks it may pose. It also investigates the accuracy that driver monitoring systems have in detecting conditions like driver distraction and drowsiness, and explores ways future drivers may respond to the broader introduction of this technology on passenger vehicles.

## The Race for More Vehicle Automation

The last decade has witnessed a steep rise in the amount of automation and assistance systems being introduced into passenger vehicles. It was 2012 when Google released the now iconic video of Steve Mahan, a legally blind person, in the driver’s seat of a fully self-driving vehicle. Since then, the reality of a (near) future where anyone could ride or even own a vehicle that is fully capable of driving itself without the need for human intervention has loomed closer and closer. In keeping with its promises, the automotive industry has released technology that just a few years prior the general public would only have dreamt of. For example, in 2015 Tesla released the first version of its “Autopilot” system. The system is capable of taking over the vehicle’s steering and acceleration with the human driver being left in charge of supervising its functioning and resuming manual control whenever necessary. In 2017, Waymo started testing its fully self-driving vehicle with real customers in the Metro Phoenix area. Since then, other manufacturers have joined the race to provide more vehicle technology to motorists and riders.

As the race for more vehicle automation was heating up, the Human Factors research community also started looking into related issues. With the role of the human driver transitioning from that of *vehicle operator* on manually-driven vehicles (or level 0 vehicles per the SAE J3216 standard; SAE, 2021) to that of *system supervisor* on vehicles capable of controlling both acceleration and steering in selected scenarios (level 2 and 3; SAE, 2021) to that of *vehicle passenger* on vehicles capable of driving without driver input (level 4 and 5; SAE, 2021), this has brought forward some less-than-unexpected problems. For decades, Human Factors researchers have explored human interaction with automation in fields like aviation and air traffic control operations. For example, early work by Parasuraman (1987) and Wiener (1973) highlighted the risk that having to sustain attention during extended monitoring tasks had for vigilance decrement and boredom. Related research by Waldrop (Waldrop, 1989a; 1989b) pointed at the then-nascent issue of how responsibilities should be shared between aircraft pilots and automated flying systems, and how the lack of adequate understanding on who-does-what may lead to lapses in performance.

## History Repeats Itself

Despite these early warnings, history proved to repeat itself. Alongside the race for more automation, recent years have also witnessed a series of quite significant setbacks. Among the most prominent was a 2018 fatal crash involving an Uber vehicle wherein an SUV, which at the time was being driven without any human input, struck and killed a pedestrian attempting to cross a street in Phoenix, AZ. Despite a backup human driver occupying the driver’s seat, it was later revealed that she was distracted watching TV on her phone (WKU, 2023). Numerous collisions have also involved vehicles which at the time of the crash were being operated in SAE level-2 mode. Having an incorrect understanding (or mental models) of these systems’ capabilities and limitations and the difficulty gauging what mode the vehicle is on (or mode confusion) have been identified as common issues when operating these systems (Cummings & Bauchwitz, 2022; Endsley, 2017; Lee & City, 2008). Safety investigations conducted following these accidents also pointed at the driver’s overreliance on the automated system as a contributing factor to the crashes, and the foreseeability of automation misuse as an issue meriting further attention (NTSB, 2020).

## Countermeasures to Driver Disengagement

The number of vehicles capable of driving in SAE level-2 mode is increasing year over year (Statista, 2022). With it, the attention from the Human Factors and regulator communities over the potential unintended consequences of operating these systems is also expected to grow. In 2021 NHTSA started requiring manufacturers of level-2 systems to report collisions involving their vehicles, and in 2022 it published an initial report documenting the number of collisions by manufacturer observed over the prior 11 months (NHTSA, 2022). Human Factors efforts have also started looking into ways to improve drivers’ use of these systems. In 2019, the American Automobile Association (AAA, 2019) issued a report recommending the standardization of the terminology for assistance and automated driving systems by manufacturers. Building on the notion that inconsistent naming leads to driver confusion (Biondi, 2020; Singer et al., 2022; Teoh, 2019), the report poses that adopting a more consistent approach that clarifies the actual capabilities and limitations of the system may reduce the potential for drivers misusing or abusing these systems.

The introduction of driver monitoring systems that are designed to detect drivers’ state and alert them should conditions like distraction or drowsiness be detected also represents a promising solution. Current systems use steering input like the torque applied on the steering wheel and steering wheel movements, and driver-facing cameras that capture head or eye movements as means to determine the driver’s physiological state and their fitness to drive. The expected safety benefits of these systems are tangible, with a recent article estimating their adoption to reduce fatalities by the thousands (Lenné, 2021). Yet, available data on the accuracy of these systems and their ability to fulfill their road safety promises is still unproven. In one available study, AAA conducted an assessment of four driver monitoring systems, two using steering input and two using driver-facing cameras (AAA, 2022). System performance was measured by how accurate the systems were in detecting conditions of driver distraction and disengagement during level-2 driving. Although none of the four systems showed a 100% accuracy, systems equipped with driver-facing cameras showed superior performance relative to systems that monitored steering input alone.

## This Special Issue

With more to be learned about driver behavior during the use of automated driving systems and little knowledge being available on the accuracy of driver monitoring systems, this special issue aims to add to our understanding of the safety risks and benefits of adopting new vehicle technology as well as some of the inherent challenges in implementing such systems. Lee et al. (2023) investigated the role that the driving style adopted by the SAE level-2 system (defensive to aggressive) had on the driver’s tendency to resume manual control upon the vehicle encountering emergency situations. Results showed clear benefits in adopting driving styles that can adapt to driver trust and road events. Biondi et al. (2023) measured vigilance decrements with drivers operating four vehicles (a Volvo, a Tesla, a Nissan, and a Cadillac) in manual and SAE level-2 mode. Driving in level-2 mode resulted in a deeper vigilance decrement relative to manual driving, and significant variability was found across vehicles based on their unique system designs. Herbers et al. (2023) used glance and vehicle metrics (speed, steering input, etc.) to develop and test the accuracy of four algorithms to detect driver distraction. Although models were approximately 50% accurate in identifying driver state in conditions without distraction, accuracy decreased to 30% for one algorithm in the high distraction conditions. Both outcomes underscore the challenges in accurately classifying distraction states—even when systems are using both driver and vehicle inputs. Mulhall et al. (2023) adopted a driver monitoring system manufactured by Seeing Machines Ltd. (Australia) to detect distraction-induced lizard (primarily gaze movements) and owl glances (primarily head movements) during naturalistic driving. Results showed that lizard glances were far more common than owl glances, indicating the need for the adoption of more sophisticated driver monitoring systems that can detect finer gaze movements for the correct classification of driver distraction. Ayas et al. (in press) conducted a scoping review on in-vehicle interventions for driver drowsiness. Whereas the work identified the metrics currently used for drowsiness detection, it also explored the issue of what the in-vehicle system should do when driver drowsiness is detected. It also raised the question of how drivers will adapt and react to the introduction of driver monitoring systems long-term. While at the current time these systems are expected to be the one solution that fixes all road safety problems, the extent to which their promised benefits translate into real-world solutions remains to be seen. Collectively, the papers included in this special section help to fill some critical gaps in our knowledge in this important area. Moreover, they underscore some of the challenges related to the design and implementation of driver state monitoring systems that bear further consideration as these systems become more commonplace.

## Key Points

- With vehicle automation, the role of the human driver is shifting from that of system operator to that of system supervisor.
- This results in a higher risk for drivers to become disengaged from the task of supervising the system functioning.
- This special issue investigates potential safety risks of vehicle automation, and the accuracy of using driver monitoring systems.

## ORCID iDs

Francesco N. Biondi https://orcid.org/0000-0002-5558-4707

Birsen Donmez https://orcid.org/0000-0002-1427-7516
